# Investigation of Dombrock Blood Group Alleles and Genotypes among Saudi Blood Donors in Southwestern Saudi Arabia

**DOI:** 10.3390/genes13061079

**Published:** 2022-06-17

**Authors:** Amr J. Halawani, Abdullah S. Mansor, Hamza M. Assaggaf, Hibah A. Almasmoum, Hisham I. Abu-Tawil, Khalaf F. Alsharif, Gasim Dobie, Mahmoud M. Habibullah

**Affiliations:** 1 Department of Laboratory Medicine, Faculty of Applied Medical Sciences, Umm Al-Qura University, Makkah 24381, Saudi Arabia; hmsaggaf@uqu.edu.sa (H.M.A.); hamasmoum@uqu.edu.sa (H.A.A.); 2 Department of Medical Laboratory Technology, Faculty of Applied Medical Sciences, Jazan University, Jazan 82911, Saudi Arabia; amansor@jazanu.edu.sa (A.S.M.); gdobie@jazanu.edu.sa (G.D.); mhabibullah@jazanu.edu.sa (M.M.H.); 3 SMIRES for Consultation in Specialized Medical Laboratories, Jazan University, Jazan 82911, Saudi Arabia; 4 Department of Laboratory and Blood Bank, Prince Mohammed bin Nasser Hospital, Ministry of Health, Jazan 82943, Saudi Arabia; hisha0707@gmail.com; 5 Department of Laboratory and Blood Bank, King Faisal Medical City for Southern Regions, Ministry of Health, Abha 62523, Saudi Arabia; 6 Department of Clinical Laboratory Science, College of Applied Medical Sciences, Taif University, P.O. Box 11099, Taif 21944, Saudi Arabia; alsharif@tu.edu.sa

**Keywords:** Dombrock blood group, blood groups, blood group genotyping, blood transfusion, Saudi Arabia

## Abstract

The Dombrock (DO) blood group system has two primary antigens, Do^a^ and Do^b^, which can cause delayed hemolytic transfusion reactions. The paucity of specific monospecific antibodies can hamper the typing based on these antigens. Thus, blood group genotyping (BGG) was investigated as a possible solution. Sequence-specific primers were designed to target a single nucleotide polymorphism (rs11276) on the *ART4* gene encoding the DO*A and DO*B alleles. Blood samples (*n* = 150) from randomly selected volunteer donors were used. DNA was extracted and resulting PCR products were purified and sequenced. The allelic frequencies of DO*A and DO*B were (*n* = 122, 40.67%) and (*n* = 178, 59.33%), respectively. The distributions of DO genotypes were as follows: DO*A/DO*A (*n* = 20), 13.33%; DO*B/DO*B (*n* = 48), 32.00%; and DO*A/DO*B (*n* = 82), 54.67%. In conclusion, this study reports on the allelic frequencies of DO*A and DO*B of the DO blood group system in Jazan Province, Kingdom of Saudi Arabia. Furthermore, this study reports on the prevalence of each genotype, of which DO*A/DO*B was the most abundant. This study contributes significantly to build the current blood donor database in Southwestern Saudi Arabia. Moreover, it may assist in providing safe blood to polytransfused patients and reduce the risk of the red cell alloimmunization.

## 1. Introduction

The Dombrock (DO) blood group system (014, International Society of Blood Transfusion) was first identified in the 1960s [1]. The anti-Do^a^ antigen was identified in 1965, and the anti-Do^b^ antigen was identified 8 years later [2,3]. Three phenotypes result from these antigens: Do(a^+^b^−^), Do(a^−^b^+^), and Do(a^+^b^+^); phenotypic frequency varies between populations [4].

In 1967, two additional antigens were independently identified, Gregory (Gy^a^) and Holly (Hy), which were identified as high-prevalence antigens in the DO blood group system [5,6]. In 1972, a third high-prevalence antigen was identified as Jo^a^ [7]. There is also a null phenotype in the Dombrock blood group system, in which all antigens are negative [8].

Dombrock antigens are displayed on a glycosylphosphatidylinositol (GPI)-linked glycoprotein [9,10]. The antigens are absent on paroxysmal nocturnal hemoglobinuria type III red blood cells, which are used to identify Dombrock antigen-specific antibodies [10]. The investigation of Dombrock antigen expression is complicated and difficult due to the scarcity of monospecific antisera and the weak reactivity [11]. The antibodies of the DO blood group system can be stimulated by blood transfusion or pregnancy. The result of the direct antiglobulin test may be positive, but it is not caused hemolytic disease of the fetus and newborn [4]. However, anti-Do^a^ and anti-Do^b^ antibodies have been reported to cause immediate and delayed hemolytic transfusion reactions (HTR) [12,13,14,15,16].

The DO*A and DO*B genes differ in only three nucleotides: two silent mutations (c.378>T, c.624T>C) and a single missense mutation (c.793A>G) that gives rise to an amino acid substitution (p.Asn265Asp) in the exon 2 of the *ART4* gene [17]. The frequencies of DO alleles and genotypes vary around the world [18]. Generally, the DO*A/DO*A genotype seems to be less prevalent compared to the other DO genotypes [4]. The DO*B/DO*B genotype is more prevalent in Japanese and Thai populations at 76.5 and 86.5%, respectively [19,20]. The heterozygous DOA*/DO*B genotype is more predominant in the Caucasian population at 46% [3]. Intriguingly, DO genotyping can be useful to distinguish the frequency and diversity of the DO alleles between different ethnic backgrounds, especially in countries hosting immigrants [21].

Saudi Arabia has a unique geographical location that is located between Asia and Africa. In past, many tribes have migrated due to religious purposes. Therefore, Saudi Arabia comprises multi-ethnicities. Currently, Saudi Arabia is divided into six regions comprising 13 provinces. The Jazan Province is endemic with sickle cell disease (SCD) and β-thalassemia [22]. Those patients may require frequent blood transfusion units and may develop alloantibodies. Therefore, the provision of compatible blood transfusions may be challenging for such patients to ensure their safety and preclude the risk of alloimmunization [23].

Various studies were carried out to demonstrate the prevalence of different blood group antigens in the southwestern Saudi Arabian population [24,25,26,27]. However, the DO blood group system remains poorly investigated in Saudi Arabia. This study aims to investigate the DO alleles, DO*A and DO*B, using the blood group genotyping (BGG) approach in blood donors from a Saudi Arabian population in the southwestern Jazan Province. Furthermore, the DO blood group genotypes are also determined for this population and compared to different ethnicities around the world.

## 2. Materials and Methods

### 2.1. Blood Samples

The study was conducted in accordance with the Declaration of Helsinki, and approved by the Jazan Hospital Institutional Review Board ((H-10-Z-068, protocol code No. 2017). Blood samples were obtained from Prince Muhammad bin Nasser Hospital in the Jazan Province of Saudi Arabia. Samples were obtained from volunteer Saudi blood donors living in the Jazan area. Samples were received in tubes containing ethylenediaminetetraacetate (EDTA). DNA was extracted using a GeneJET Whole Blood Genomic DNA Purification Mini Kit (Thermo Fisher Scientific, Paisley, UK) according to the manufacturer’s instructions. Purified DNA was assessed for quality and quantity using a NanoDrop 200 spectrophotometer (Thermo Fisher Scientific, Paisley, UK).

### 2.2. PCR and Sequencing

A new primer pair was designed to capture the single nucleotide variant (SNV), ID: rs11276, using the National Center for Biotechnology Information primer BLAST tool (Table 1). The target amplicon size was 538 base pairs (bp). Primers were provided by Macrogen (Seoul, South Korea) with high-performance liquid chromatography purification. PCR reactions contained 1× Phusion Green Host Start II High-Fidelity PCR Master Mix (Thermo Fisher Scientific, Paisley, UK), 50 ng of DNA template, and 0.5 µM of each forward and reverse primer. Cycling conditions were as follows: initial denaturation at 98 °C for 30 s, followed by 35 cycles of denaturation 98 °C for 10 s; annealing at 60 °C for 30 s; and extension at 72 °C for 30 s, and final extension at 72 °C for 10 min followed by 4 °C hold. PCR amplicons were visualized using 2% agarose gel electrophoresis. PCR samples were sent to Macrogen (Seoul, South Korea) for sequencing services. The MacVector Software, Version 12.7 (MacVector, Inc., Apex, NC, USA) was used to visualize sequencing electropherograms.

### 2.3. Statistical Analysis

The sample size was calculated using G*Power software Version 3.1.9.4 with a two-sided exact test for one proportion. This sample size had satisfactory power to detect the significant difference between the estimated and observed population prevalence with a 5% level of significance, 11% allowable margin of error, and 81% power in which a ‘null value’ of 0.66 was used. The outcome was estimated to be a total of 143 samples and it was rounded up to 150 samples in the current study.

The frequency of DO alleles was identified and standardized as percentages. A chi-squared test was used in order to compare the frequencies of DO alleles in the Saudi population who live in the Jazan Province with those of the other reported ethnicities. *p*-values < 0.01 were indicative of highly significant differences.

## 3. Results

The frequencies of the DO alleles are listed in Table 2. The frequency of the DO*A allele was 40.67%, while that of the DO*B allele was 59.33%. Table 3 lists the prevalence of the genotypes and their predicted phenotypes in the DO blood group system. The prevalence of the DO*A/DO*A and DO*B/DO*B homozygous genotypes were 13.33 and 32.00%, respectively. Interestingly, the most common genotype was the heterozygous DO*A/DO*B, with a prevalence of 54.67%. The prevalence of DO genotypes and their predicted phenotypes in the population in the present study was compared to that of other ethnicities [3,4,19,20], as shown in Table 4.

Samples with the DO*A/DO*A genotype had a missense variant c.793G>A (rs11276), which encoded p.Asp265Asn of the translated protein. On the other hand, the DO*B/DO*B genotype observed (c.793A>G) encoding p.Asn265Asp. Both A and G nucleotides at the same position were observed in the group with the heterozygous genotype (DO*A/DO*B). Furthermore, a synonymous SNV (rs3088189) was investigated, in which c.624C>T was associated with the DO*A allele and c.624T>C with the DO*B allele. The encoded amino acid remained unchanged (p.Leu208=) [28].

## 4. Discussion

Comprehensive knowledge regarding the frequency of blood group alleles/antigens and genotypes/phenotypes is crucial in transfusion practice. Several studies have previously been conducted in the Jazan Province of Saudi Arabia to investigate the prevalence of different blood groups to build a blood donor database [24,25,26,27]. The data reported here may assist in reducing the possibility of alloimmunization incidents, particularly in patients requiring multiple transfusions, by assisting in the selection of appropriate blood units [29].

To the best of our knowledge, this is the first study from the Kingdom of Saudi Arabia to report the frequencies of the primary alleles of the DO blood group system, using genotyping by sequencing. The frequency of the DO*A and DO*B alleles in the Saudi Arabian population living in the Jazan Province were 40.67% and 59.33%, respectively. Relatively similar frequencies were reported in the 1000 genomes project for the DO*A allele in different ethnic backgrounds as follows: 38.50% in South Asians, 37.30% in Europeans, and 37.50% in Americans. However, the DO*A allele differed in Southwestern Saudis from that of populations of other ethnicities (26.90% in African and 9.80% in East Asians) [18]. The DO*B allele was different to what was reported for both African and East Asian populations at 73.10% and 90.20%, respectively [18].

The heterozygous genotype DO*A/DO*B in the present study was the most prevalent genotype at 54.67%. Interestingly, the Saudi Arabian population exhibited the highest prevalence of the heterozygous genotype, as compared to that of other ethnicities as shown in Table 4. Similar statistical results were observed in other DO genotypes when comparing the Jazan population with African, ASW, PUR, BEB, and Caucasian populations [3,4,18]. Conversely, there were significant differences in the frequencies of DO genotypes between the Jazan population and FIN (*p* < 0.05), CHS (*p* < 0.01), Japanese (*p* < 0.01), and Thai groups (*p* < 0.01) [18,19,20].

The paucity of serological reagents for detecting the DO blood group antigens can obstruct phenotyping. However, BGG can be used to resolve such situations especially when introducing a high-throughput platform and including DO alleles, such as the microarray approach [30]. DO BGG may help to reduce the alloimmunization risk for patients undergoing multiple transfusions, including those with sickle cell disease and thalassemia [31]. Furthermore, BGG can be useful for the high-throughput screening of donor and patient samples, by facilitating the development of a donors’ database and assisting in the selection of compatible blood units when seeking to prevent HTR [32].

One limitation of this study was that the designed sequencing primers were unable to target a SNV c.378C>A, which encodes a silent mutation (p.Tyr126=). Another limitation was that other alleles belonging to the DO blood group system, such as (Hy, Gy^a^, and Jo^a^), were not investigated. However, the DO*A and DO*B alleles have been reported to be more clinically significant as compared to the other DO alleles [16,33].

## 5. Conclusions

In summary, this study investigated the frequency of the primary alleles of the DO blood group system, DO*A and DO*B, in a population from the Jazan Province of the Kingdom of Saudi Arabia. Furthermore, DO genotypes and their predicted phenotypes were determined and the heterozygous status was the most prevalent genotype (DO*A/DO*B). DO genotypes were similar to the African and Caucasian populations and were different compared to CHS, FIN, Japanese, and Thai groups. Crucially, a significant contribution has been added to the current blood donor database in the Jazan Province. This may ensure the safety of the provided blood to polytransfused patients, including SCD and β-thalassemia patients, and reduce the risk of red cell alloimmunization.

## Figures and Tables

**Table 1:** DO primers used for sequencing.

Primer	5′ to 3′ Sequence	Product Size (bp)	Chromosomal Location
Do-rs11276-F	ACACACGCTGTGGCTATTTTG	538	chr12:14840409-14840946
Do-rs11276-R	GTGATCCTGAGTGGCCTCAAT		

**Table 2:** The frequency of DO alleles in Saudi blood donors from the Jazan Province.

Allele	Observation (n)	Frequency (%)
DO*A	122	40.67
DO*B	178	59.33

**Table 3:** The frequencies of the DO genotypes and their predicted phenotypes in a population of Saudi blood donors from the Jazan Province.

DO Allele	Genotype	Predicted Phenotype	Observation (n)	Frequency (%) *n* = 150
DO*A	DO*A/DO*A	Do(a^+^b^−^)	20	13.33
DO*A and DO*B	DO*A/DO*B	Do(a^+^b^+^)	82	54.67
DO*B	DO*B/DO*B	Do(a^−^b^+^)	48	32

**Table 4:** Comparison of frequencies of DO phenotypes between multiple ethnic groups and the population evaluated in the present study.

Population	DO*A/DO*A	DO*A/DO*B	DO*B/DO*B	*p*-Values
Southwestern Saudis (Current study)	13.33	54.67	32	-
African [4]	11	44	45	0.17 (>0.05)
Japanese [19]	1.50	22	76.50	0.00 (<0.01) **
Thai [20]	0.50	13	86.50	0.00 (<0.01) **
Caucasian [3]	16	46	31	0.65 (>0.05)
ASW	9.80	42.60	47.50	0.08 (>0.05)
PUR	18.30	50	31.70	0.51 (>0.05)
CHS	0.00	18.10	81.90	0.00 (<0.01) **
FIN	8.10	38.40	53.50	0.01 (<0.05) *
BEB	11.60	52.30	36	0.84 (>0.05)

ASW: African ancestry in Southwest United States; PUR: Puerto Rican in Puerto Rico; CHS: Southern Han Chinese; FIN: Finnish in Finland; BEB: Bengali in Bangladesh. * Significant (*p* < 0.05); ** Highly significant (*p* < 0.01).

## Data Availability

Not applicable.
